# Does interferon-free direct-acting antiviral therapy for hepatitis C after curative treatment for hepatocellular carcinoma lead to unexpected recurrences of HCC? A multicenter study by the Japanese Red Cross Hospital Liver Study Group

**DOI:** 10.1371/journal.pone.0194704

**Published:** 2018-04-16

**Authors:** Toshie Mashiba, Kouji Joko, Masayuki Kurosaki, Hironori Ochi, Yukio Osaki, Yuji Kojima, Ryo Nakata, Tohru Goto, Akahane Takehiro, Hiroyuki Kimura, Akeri Mitsuda, Chiharu Kawanami, Yasushi Uchida, Chikara Ogawa, Atsunori Kusakabe, Ryuichi Narita, Yasushi Ide, Takehiko Abe, Keiji Tsuji, Tadashi Kitamura, Kazuhiko Okada, Tetsuro Sohda, Masaya Shigeno, Takashi Satou, Namiki Izumi

**Affiliations:** 1 Center for Liver-Biliary-Pancreatic Disease, Matsuyama Red Cross Hospital, Ehime, Japan; 2 Department of Gastroenterology and Hepatology, Musashino Red Cross Hospital, Tokyo, Japan; 3 Department of Gastroenterology and Hepatology, Osaka Red Cross Hospital, Osaka, Japan; 4 Department of Hepatology, Japanese Red Cross Ise Hospital, Mie, Japan; 5 Department of Gastroenterology, Japanese Red Cross Medical Center, Tokyo, Japan; 6 Department of Gastroenterology, Omori Red Cross Hospital, Tokyo, Japan; 7 Department of Gastroenterology, Ishinomaki Red Cross Hospital, Miyagi, Japan; 8 Department of Gastroenterology, Japanese Red Cross Kyoto Daiichi Hospital, Kyoto, Japan; 9 Department of Internal Medicine, Japanese Red Cross Tottori Hospital, Tottori, Japan; 10 Department of Gastroenterology, Otsu Red Cross Hospital, Shiga, Japan; 11 Department of Gastroenterology, Matsue Red Cross Hospital, Shimane, Japan; 12 Department of Gastroenterology, Takamatsu Red Cross Hospital, Kagawa, Japan; 13 Department of Gastroenterology, Japanese Red Cross Nagoya Daini Hospital, Aichi, Japan; 14 Department of Gastroenterology, Oita Red Cross Hospital, Oita, Japan; 15 Department of Internal Medicine, Karatsu Red Cross Hospital, Saga, Japan; 16 Department of Gastroenterology, Maebashi Red Cross Hospital, Gunma, Japan; 17 Department of Gastroenterology, Hiroshima Red Cross Hospital & Atomic-bomb Survivors Hospital, Hiroshima, Japan; 18 Department of Gastroenterology, Japanese Red Cross Shizuoka Hospital, Shizuoka, Japan; 19 Department of Gastroenterology, Toyama Red Cross Hospital, Toyama, Japan; 20 Hepatology Division, Japanese Red Cross Fukuoka Hospital, Fukuoka, Japan; 21 Department of Gastroenterology, Japanese Red Cross Nagasaki Genbaku Hospital, Nagasaki, Japan; 22 Department of Gastroenterology, Nasu Red Cross Hospital, Tochigi, Japan; National Taiwan University Hospital, TAIWAN

## Abstract

**Background and aim:**

This study aimed to elucidate whether interferon (IFN)-free direct-acting antiviral (DAA) therapy for hepatitis C after curative treatment of hepatocellular carcinoma (HCC) promotes HCC recurrence in a real-world large-scale cohort.

**Methods:**

This multicenter study was conducted by the Japanese Red Cross Hospital Liver Study Group. This retrospective study analyzed 516 patients who underwent antiviral treatment for hepatitis C with either IFN (n = 148) or IFN-free DAA (n = 368) after curative HCC treatment; 78 IFN-treated patients and 347 IFN-free DAA-treated patients achieved sustained virological response (SVR). The recurrence rate of HCC was compared between the antiviral therapies. Logistic analysis and Cox proportional hazards analysis identified factors associated with early recurrence of HCC within 24 weeks of antiviral therapy and recurrence throughout the observation period, respectively.

**Results:**

AFP at the completion of antiviral therapy, clinical stage of HCC, and non-SVR were independent factors associated with early recurrence of HCC. Among patients who had achieved SVR, the clinical stage of HCC and the level of AFP at completion of antiviral therapy were independent factors associated with early recurrence of HCC. For recurrence throughout the observation period in SVR patients, AFP at completion of antiviral therapy, duration between last HCC treatment to antiviral therapy, and the number of treatments were independent factors. There was no significant difference in the rate of early recurrence of HCC or recurrence throughout the observation period between IFN and IFN-free DAA treated patients.

**Conclusions:**

There were no differences in the early recurrence rate of HCC between patients who underwent IFN and those who underwent IFN-free DAA as antiviral therapies.

## Introduction

Since the era in which interferon (IFN) was the standard treatment for hepatitis C, attempts have been made to eliminate hepatitis C virus (HCV) following hepatocellular carcinoma (HCC) treatment, but it could be achieved in only a limited number of patients due to side effects. Recently, dramatic progress has been made in anti-HCV therapies. It is now feasible to achieve a sustained virological response (SVR) rate of ≥95% even in older patients or cirrhosis patients with IFN-free direct-acting antiviral (DAA) therapy that has minimal side effects in older patients and in cirrhosis patients [1], and antiviral therapy can now be performed easily in patients who have undergone HCC treatment. It has been reported that the elimination of HCV with IFN after curative treatment for HCV-associated HCC suppresses HCC recurrences [2–5]. We have also found that it dramatically extends patients’ survival [6]. However, whether a similar effect can be achieved with DAA therapy is unclear. Furthermore, potential increases in unexpected early recurrence of HCC after HCV elimination have been reported with DAA therapy [7,8]. With this background, the HCC recurrence rate was compared between those who underwent IFN therapy and those who underwent IFN-free therapy after HCC treatment in a large-scale cohort.

## Methods

### Patients

This was a multicenter study conducted by the Japanese Red Cross Hospital Liver Study Group consisting of 22 hospitals. This study investigated 516 patients who underwent antiviral therapy for hepatitis C after curative treatment for HCC. Antiviral therapy was given from 1999 to 2016, and all patients were followed for at least 24 weeks after antiviral therapy. The study was registered in UMIN (no. 000014971), and the research protocol was approved by the Ethics Review Board (Institutional Ethics Review Committee of Matsuyama Red Cross Hospital approval number: 422) of all participating institutions. All medications were open-label drugs. All parts of the research were conducted in accordance with the principles in the Declaration of Helsinki.

### Antiviral treatment

Of the 516 patients, 148 were treated by an IFN-containing regimen, and 368 were treated by an IFN-free DAA regimen. The IFN-containing regimen included IFN monotherapy (n = 6), Peg-IFN monotherapy (n = 24), IFN plus ribavirin (RBV) (n = 9), peg-IFN plus RBV (n = 57), and peg-IFN plus RBV plus protease inhibitor such as telaprevir, simeprevir, or vaniprevir (n = 52). The IFN-free DAA regimen included asunaprevir (ASV) plus daclatasvir (DCV) (n = 159), sofosbuvir (SOF) plus RBV (n = 51), paritaprevir/ombitasvir/ritonavir (n = 7), and SOF/ledipasvir (LDV) (n = 151). The mean follow-up period after antiviral therapy was 25.5 months for the IFN group and 7.7 months for the IFN-free DAA group.

### Analysis of recurrence

Factors associated with early recurrence that appeared within 24 weeks of completing antiviral therapy were assessed in all registered patients. An identical analysis was also conducted in the subgroup of patients who achieved SVR. Furthermore, in SVR patients, recurrence during the entire observation period was analyzed, and the difference in the recurrence rate between the IFN group and the IFN-free DAA group was analyzed.

### Statistical analysis

JMP ver.13.1.0 was used for statistical analysis, and the Mann-Whitney U test and the χ^2^ test were used for comparisons between two groups. Logistic regression analysis was used to identify risk factors for early recurrence (within a half-year). A Cox proportional hazards model was used to extract risk factors for recurrence with no restrictions on the follow-up period. The Kaplan-Meier method was used for the HCC recurrence curves, and the log-rank test was used to compare groups. A value of p < 0.05 was considered to indicate a significant difference.

## Results

### Patients’ characteristics

**Fig 1:**
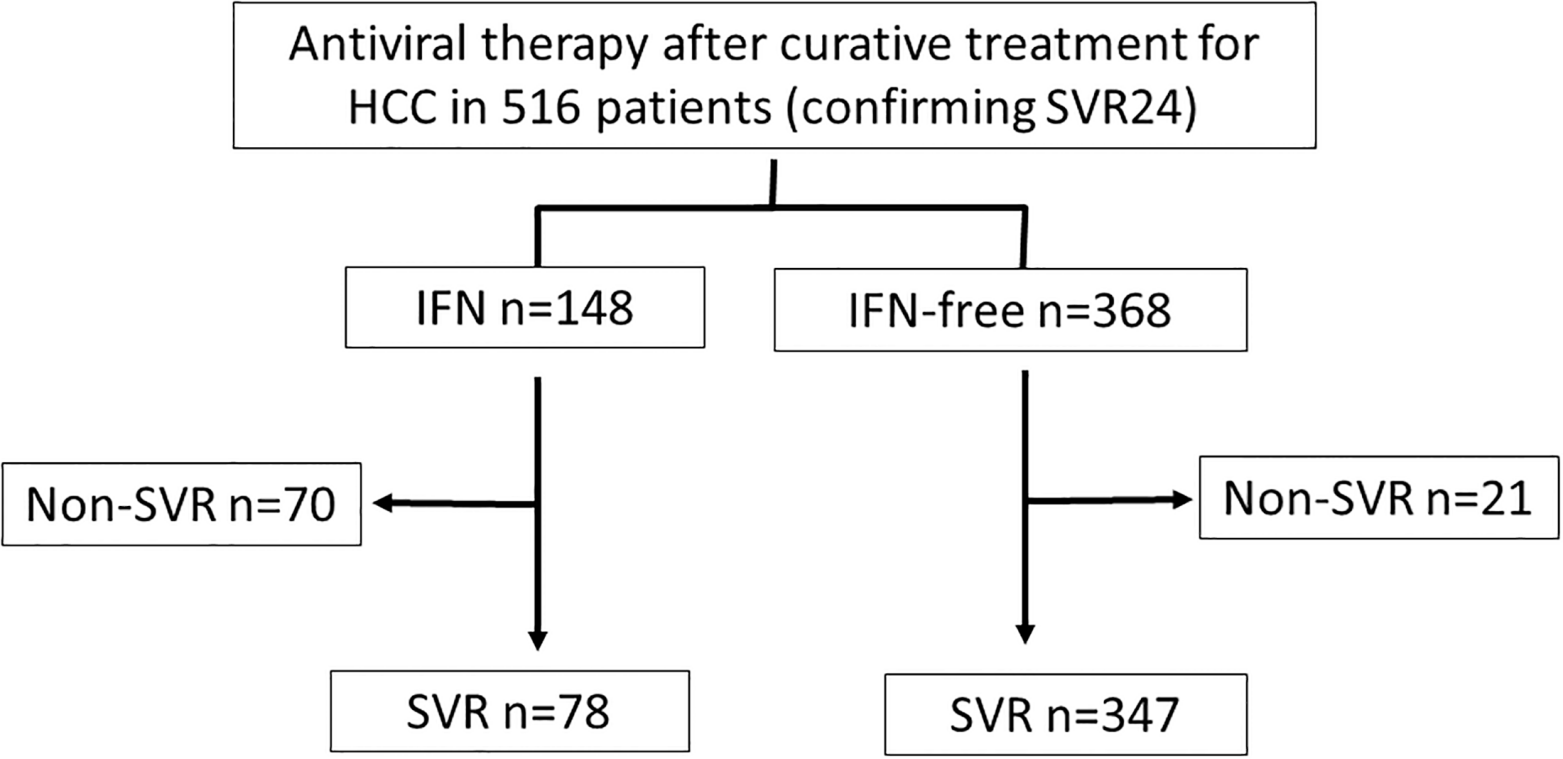
Flow sheet summarizing study selection.

The IFN-free DAA group was older, consisted of more women, and had longer duration from the date of last HCC treatment to antiviral therapy, lower leukocyte count, lower hemoglobin level, lower platelet count, lower albumin level, and higher Fib-4 index. However, data that reflect the state of HCC, such as HCC stage and AFP level, were not different, and all other clinical data were also not different (Table 1). SVR was achieved in 78/148 patients (52.7%) in the IFN group and 347/368 patients (95.4%) in the IFN-free DAA group (Fig 1).

**Table 1:** Baseline patient characteristics.

	IFN group (n = 148)	IFN-free group (n = 368)	p value
Age (years) [median (IQR)]	66 (43–80)	73 (48–88)	<0.0001
Male:Female	106:42	208:160	0.0013
Number of days from previous HCC treatment to starting antiviral therapy [median (range)]	187 (4–3348)	333 (15–5038)	<0.0001
HCC Stage (I:II:III)	52:45:19	153:102:30	0.1585
Leukocyte count (/μL) [median (IQR)]	4420 (3635–5180)	3925(3100–5100)	0.0018
Hemoglobin (g/mL) [median (IQR)]	13.8 (12.6–14.7)	12.8 (11.6–14.0)	<0.0001
Platelet count (x10^4^/μL) [median (IQR)]	12.5 (9.9–15.9)	10.2 (7.2–13.4)	<0.0001
ALT (IU/L) [median (IQR)]	53 (33–74)	44 (28–66)	0.0061
Bilirubin (mg/dL) [median (IQR)]	0.8 (0.6–1.1)	0.8 (0.6–1.1)	0.9447
Protrombin time (%) [median (IQR)]	84 (72–96)	83 (73–92)	0.7745
Albumin (g/dL) [median (IQR)]	4.0 (3.7–4.3)	3.8 (3.5–4.1)	0.0076
Fibrosis-4 index [median (IQR)]	3.8 (2.6–5.6)	5.6 (3.8–8.7)	<0.0001
α-fetoprotein (ng/mL) [median (IQR)]	10 (6–20)	10 (5–23)	0.7550

IFN: interferon, IQR: interquartile range, HCC: hepatocellular carcinoma, ALT: alanine aminotransferase.

### Early recurrence of HCC

Factors associated with early recurrence that appeared within 24 weeks of completing antiviral therapy were analyzed in all registered patients. Univariate analysis showed that the type of antiviral therapy (IFN treatment or IFN-free DAA therapy), albumin, PT, AST, AFP at completion of antiviral therapy, duration from last HCC treatment to starting antiviral therapy, number of past treatments for HCC, clinical stage of HCC, and SVR status were significant factors. These factors were then subjected to multivariate analysis, which identified AFP at completion of antiviral therapy, clinical stage of HCC, and SVR status as independent factors. IFN-free DAA therapy was not identified as an independent factor that contributed to early recurrence within 24 weeks of antiviral therapy (Table 2).

**Table 2:** Factors associated with early recurrence of HCC within six months of completing antiviral therapy.

Factor	Univariate analysis		Multivariate analysis	
	Odds ratio (95%CI)	p value	Odds ratio (95%CI)	p value
Age (years)	0.993 (0.969–1.018)	0.5826		
Sex [Male]	1.010 (0.662–1.551)	0.9639		
IFN therapy [Yes]	1.544 (0.999–2.374)	0.0487		
Previous treatment [Yes]	1.190 (0.746–1.906)	0.4672		
Leukocyte count (/μL)	1.000 (1.000–1.000)	0.1014		
Hemoglobin (g/mL)	0.988 (0.884–1.106)	0.8348		
Platelet count (x10^4^/μL)	0.959 (0.917–1.000)	0.0589		
Albumin (g/dL) <3.5 vs. ≥3.5 [<3.5]	0.565 (0.338–0.935) 1.775 (1.051–2.965)	0.0274 0.0323		
Prothrombin time (%) <70 vs. ≥70 [<70]	0.981 (0.964–0.998) 2.057 (1.137–3.666)	0.0282 0.0176		
AST (IU/L) <63 vs. ≥63 [≥63]	1.008 (1.002–1.015) 1.729 (1.126–2.653)	0.0121 0.0125		
ALT (IU/L)	1.005 (0.999–1.012)	0.0854		
AFP; α-fetoprotein (ng/mL)	1.003 (1.000–1.006)	0.0634		
DCP; des-γ-carboxy prothrombin (mAU/mL)	1.003 (0.999–1.007)	0.1533		
Fibrosis-4 index	1.038 (0.995–1.085)	0.0878		
HCV-RNA (LogIU/mL)	0.982 (0.745–1.312)	0.9014		
ALT (end of treatment)	0.999 (0.990–1.004)	0.6839		
AFP (end of treatment) <9 vs. ≥9 [≥9]	1.024 (1.012–1.040) 3.458 (2.186–5.504)	0.0003 <0.0001	1.021 (1.005–1.043) 3.487 (1.762–6.903)	0.0321 0.0003
Number of days from HCC treatment to starting antiviral therapy	0.999 (0.999–1.000)	0.0062		
Number of treatments [≥2]	2.186 (1.399–3.440)	0.0006		
Number of tumors [>3]	1.742 (0.669–4.285)	0.2346		
Tumor size [>3 cm]	1.386 (0.643–2.854)	0.3867		
Stage [III]	2.458 (1.303–4.591)	0.0049	2.526 (1.047–6.096)	0.0392
SVR [No]	0.411 (0.254–0.668)	0.0003	3.943 (1.534–10.135)	0.0044

HCC: hepatocellular carcinoma, IFN: interferon, AST: aspartate aminotransferase, ALT: alanine aminotransferase, AFP: α-fetoprotein, DCP: des-γ-carboxy prothrombin, SVR: sustained virological response.

Analysis was repeated after obtaining a cutoff value for items that showed a significant difference with continuous variables. The area under the receiver operator characteristic curve (AUROC) for each item is shown in Table 3.

**Table 3:** AUROC for items for which cutoff values were set in Table 2.

Factor [Cutoff]	AUROC
Albumin (g/dL) [3.5]	0.57547
Prothrombin time (%) [70]	0.56937
AST (IU/L) [63]	0.56816
AFP (end of treatment) (ng/mL) [9]	0.65736

### Early recurrence of HCC in the SVR subgroup

Since there was a large difference in the SVR rate between the IFN group and the IFN-free DAA group, factors associated with early recurrence within 24 weeks of completing antiviral therapy after HCC curative therapy were analyzed within the subgroup of patients who achieved SVR. Univariate analyses showed that albumin, PT, AST, Fib-4 index, AFP at completion of antiviral therapy, duration from last HCC treatment to starting antiviral therapy, number of past treatments for HCC, clinical stage of HCC, and number of HCC nodules were significant. However, the type of antiviral therapy (IFN treatment or IFN-free DAA therapy) was not a significant factor. Factors that were significant on univariate analysis were subjected to multivariate analysis, which identified clinical stage of HCC and the number of treatments as independent factors (Table 4).

**Table 4:** Factors associated with early recurrence of HCC within six months of completing antiviral therapy in patients who achieved SVR.

Factor	Univariate analysis		Multivariate analysis	
	Odds ratio (95%CI)	p value	Odds ratio (95%CI)	p value
Age (years)	1.004 (0.975–1.034)	0.8055		
Sex [Male]	1.030 (0.629–1.706)	0.9064		
IFN therapy [Yes]	1.023 (0.553–1.824)	0.9409		
Previous treatment [Yes]	1.036 (0.614–1.747)	0.8941		
Leukocyte count (/μL)	1.000 (1.000–1.000)	0.2433		
Hemoglobin (g/mL)	0.967 (0.850–1.103)	0.6150		
Platelet count (x10^4^/μL)	0.970 (0.921–1.017)	0.2240		
Albumin (g/dL) <3.6 vs. ≥3.6 [<3.6]	0.542 (0.300–0.968) 2.041 (1.181–3.505)	0.0398 0.0109		
Prothrombin time (%) <70 vs. ≥70 [<70]	0.971 (0.951–0.990) 2.677 (1.401–5.042)	0.0042 0.0032		
AST (IU/L) <63 vs. ≥63 [≥63]	1.010 (1.002–1.018) 2.177 (1.313–3.608)	0.0176 0.0026		
ALT (IU/L)	1.003 (0.996–1.011)	0.3156		
AFP (ng/mL)	1.002 (0.999–1.005)	0.1370		
DCP (mAU/mL)	1.006 (1.001–1.014)	0.0733		
Fibrosis-4 index	1.031 (0.983–1.080)	0.1899		
HCV-RNA (LogIU/mL)	0.927 (0.686–1.270)	0.6260		
ALT (end of treatment)	0.996 (0.979–1.004)	0.4914		
AFP (end of treatment)	1.018 (1.006–1.034)	0.0114		
<9 vs. ≥9 [≥9]	2.667 (1.559–4.560)	0.0004	2.340 (1.169–4.888)	0.0170
Number of days from HCC treatment to starting antiviral therapy	0.999 (0.999–1.000)	0.0061		
Number of treatments [≥2]	2.026 (1.202–3.451)	0.0085		
Number of tumors [>3]	2.010 (0.667–5.537)	0.1879		
Tumor size [>3 cm]	1.555 (0.642–3.511)	0.3033		
Stage [III]	2.289 (1.086–4.686)	0.0254	3.089 (1.241–7.692)	0.0154

HCC: hepatocellular carcinoma, SVR: sustained virological response, IFN: interferon, AST: aspartate aminotransferase, ALT: alanine aminotransferase, AFP: α-fetoprotein, DCP: des-γ-carboxy prothrombin.

The AUROC for each item is shown in Table 5.

**Table 5:** AUROC for items for which cutoff values were set in Table 4.

Factor [Cutoff]	AUROC
Albumin (g/dL) [3.6]	0.58211
Prothrombin time (%) [70]	0.60711
AST (IU/L) [63]	0.58842
AFP (end of treatment) (ng/mL) [9]	0.61189

### Recurrence of HCC throughout the observation period in the SVR subgroup

**Fig 2:**
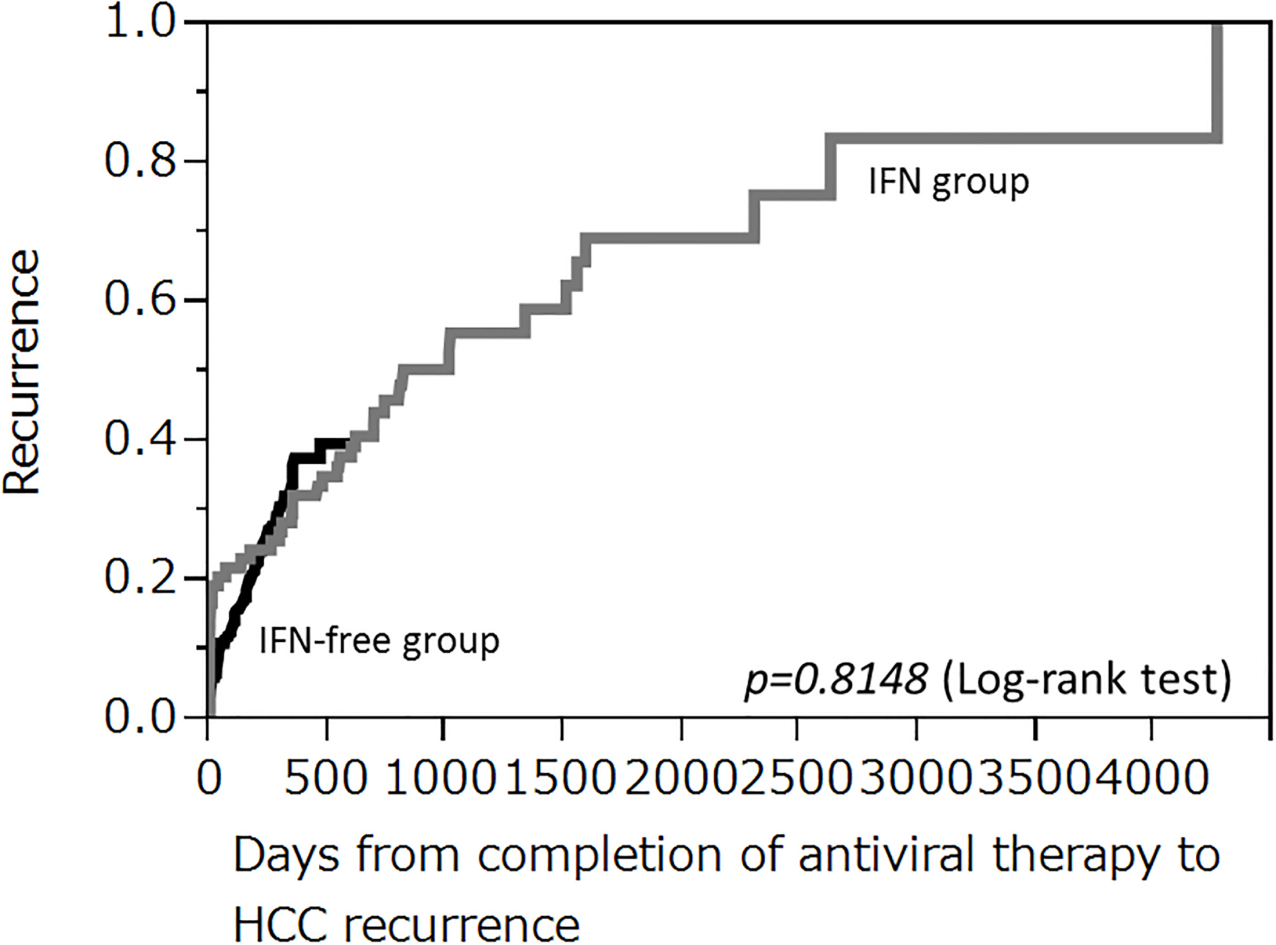
Cumulative hepatocellular carcinoma (HCC) recurrence rate in patients who achieved sustained virological response. Kaplan-Meier analysis does not show a significant difference between the IFN group (gray line) and IFN-free DAA group (black line).

**Fig 3:**
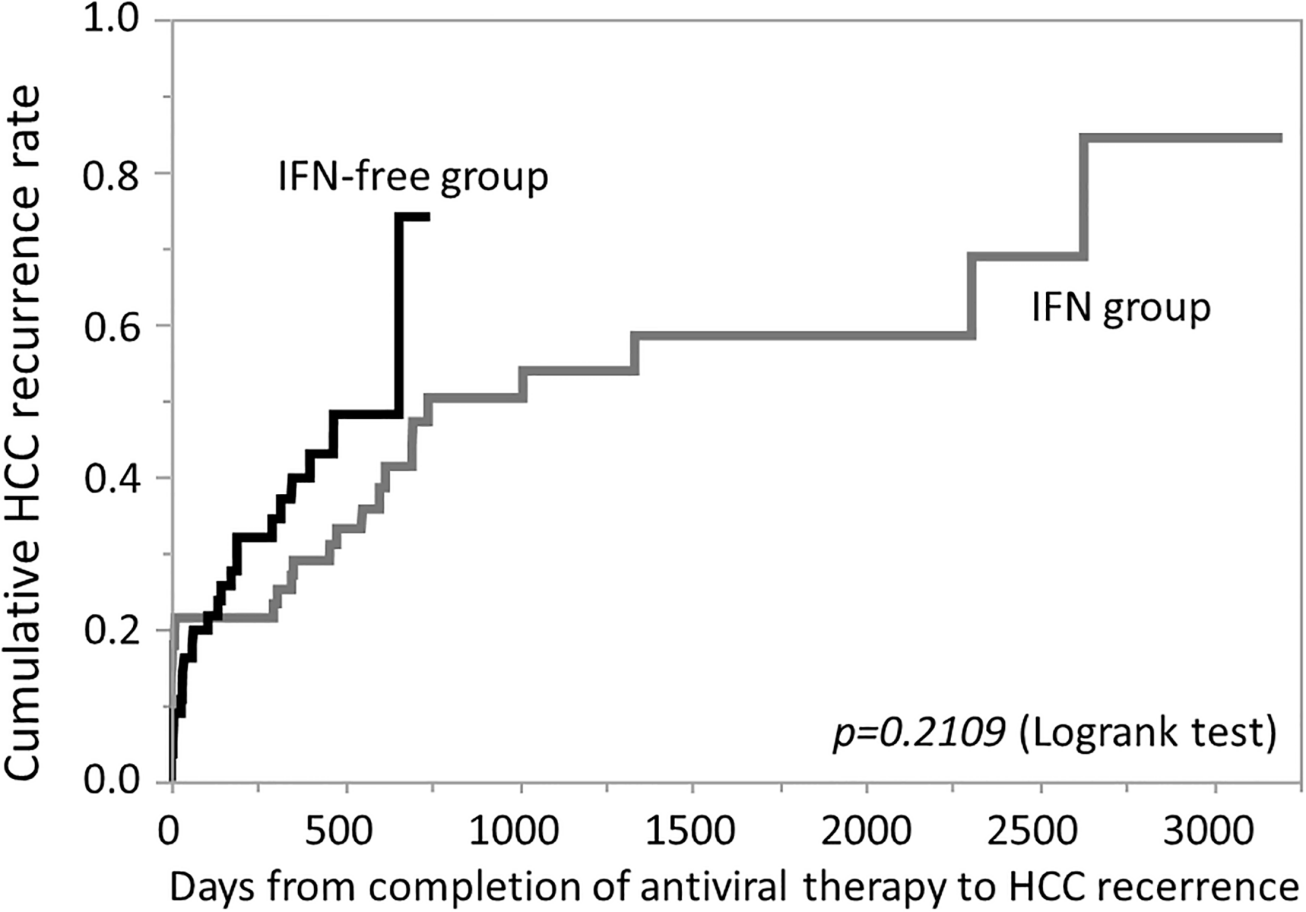
HCC cumulative recurrence rate after propensity score matching. Kaplan-Meier analysis performed after matching also revealed no significant difference between the IFN group (gray line) and the IFN-free DAA group (black line).

Factors associated with recurrence throughout the observation period, without restricting to 24-week early recurrence, were analyzed in SVR patients. Univariate analysis showed that platelets, albumin, PT, AST, ALT, Fib-4 index, AFP at completion of antiviral therapy, duration from last HCC treatment to starting antiviral therapy, number of past treatments for HCC, and number of HCC nodules were significant. However, the type of antiviral therapy (IFN treatment or IFN-free DAA therapy) was not a significant factor. On multivariate analysis, AFP at completion of antiviral therapy and duration from last HCC treatment to starting antiviral therapy were identified as independent factors (Table 6). The AUROC for each item is shown in Table 7. Kaplan-Meier analysis also did not demonstrate a significant difference between the IFN group and the IFN-free DAA group (Fig 2). Propensity scores were calculated with age, sex, FIB4, HCC stage, and the number of days from HCC treatment until the start of antiviral treatment as covariates, and propensity score matching (1:1) was performed (Table 8). No differences were seen at the time in a comparison between the 2 groups of the HCC cumulative recurrence rate (Fig 3).

**Table 6:** Factors associated with recurrence of HCC after completing antiviral therapy in patients who achieved SVR.

Factor	Univariate analysis		Multivariate analysis	
	Hazard ratio (95%CI)	p value	Hazard ratio (95%CI)	p value
Age (years)	1.001 (0.981–1.022)	0.9136		
Sex [Male]	1.120 (0.791–1.605)	0.5276		
IFN therapy [Yes]	0.950 (0.605–1.445)	0.8148		
Previous treatment [Yes]	0.908 (0.629–1.308)	0.6057		
Leukocyte count (/μL)	1.000 (1.000–1.000)	0.4530		
Hemoglobin (g/mL)	0.977 (1.068–1.024)	0.6048		
Platelet count (x10^4^/μL) <10 vs. ≥10 [<10]	0.964 (0.931–0.997) 1.563 (1.035–2.361)	0.0333 0.0337		
Albumin (g/dL) <3.6 vs. ≥3.6 [<3.6]	0.558 (0.376–0.830) 1.717 (1.080–2.723)	0.0041 0.0226		
Prothrombin time (%) <70 vs. ≥70 [<70]	0.974 (0.961–0.988) 2.683 (1.513–4.759)	0.0002 0.0008		
AST (IU/L) <63 vs. ≥63 [≥63]	1.010 (1.005–1.014) 2.472 (1.603–3.827)	0.0002 0.0008		
ALT (IU/L) <55 vs. ≥55 [55]	1.007 (1.002–1.011) 1.895 (1.247–2.881)	0.0059 0.0028		
AFP (ng/mL)	1.001 (1.000–1.003)	0.1223		
DCP (mAU/mL)	1.001 (0.999–1.002)	0.4164		
Fibrosis-4 index	1.036 (1.011–1.056)	0.0068		
HCV-RNA (LogIU/mL)	0.961 (0.793–1.183)	0.6997		
ALT (end of treatment)	1.000 (0.996–1.002)	0.8877		
AFP (end of treatment)	1.001 (1.000–1.001)	0.0141		
<9 vs. ≥9 [≥9]	3.428 (2.148–5.500)	<0.0001	2.165 (1.305–3.564)	0.0030
Number of days from HCC treatment to starting antiviral therapy	0.999 (0.999–1.000)	0.0097	0.999 (0.999–1.000)	0.0270
Number of treatments [≥2]	1.757 (1.237–2.506)	0.0138	1.622 (1.012–2.617)	0.0444
Number of tumors [>3]	2.221 (1.119–3.981)	0.0466		
Tumor size [>3 cm]	1.076 (0.596–1.821)	0.7983		
Stage [III]	1.687 (0.996–2.710)	0.0517		

HCC: hepatocellular carcinoma, SVR: sustained virological response, IFN: interferon, AST: aspartate aminotransferase, ALT: alanine aminotransferase, AFP: α-fetoprotein, DCP: des-γ-carboxy prothrombin

**Table 7:** AUROC for items for which cutoff values were set in Table 6.

Factor [Cutoff]	AUROC
Platelet count (x10^4^/μL) [<10]	0.56172
Albumin (g/dL) [3.6]	0.56000
Prothrombin time (%) [70]	0.61047
AST (IU/L) [63]	0.60616
ALT (IU/L) [55]	0.57519
AFP (end of treatment) (ng/mL) [9]	0.64714

**Table 8:** Patient characteristics after propensity score matching.

	IFN group (n = 56)	IFN-free group (n = 56)	p value
Age (years)	66.0±6.7	66.6±9.1	0.7158
Male:Female	42:14	41:15	0.8292
Number of days from previous HCC treatment to starting antiviral therapy [median (range)]	194 (41–2667)	142 (22–1630)	0.0750
HCC Stage (I : II : III)	21 : 26 : 9	22 : 27 : 7	0.8638
Fibrosis-4 index [median ± IQR]	3.77±1.86	3.79±1.34	0.4236

## Discussion

In the present study using a multicenter study cohort, there was no difference in the early recurrence rate of HCC between patients who underwent IFN and those who underwent IFN-free DAA as antiviral therapies after curative HCC treatment. This is in contrast with the previous reports that described potential increases in unexpected early recurrence of HCC after HCV elimination with DAA therapy [7,8]. The present data have a strength in that they were derived from a multicenter study that reflects the real-world data in Japan.

The SVR rate was as high as 94.3% in the IFN-free DAA-treated patients who had previously undergone HCC treatment. There is no doubt that IFN-free DAA therapy could be the standard antiviral treatment in terms of viral cure. However, the question remains whether DAA therapy in patients with a past history of HCC could be justified in terms of cost-effectiveness or improvement of survival. One of the major concerns is that the suppression of de novo HCC or recurrence of HCC may be weaker in IFN-free DAA regimens than in IFN-containing regimens.

In contrast to DAA that simply blocks HCV replication, treatments containing IFN are supposed to suppress HCV replication, to eliminate HCV-infected hepatocytes via immuno-stimulation, and to have an anti-tumor effect [9]. Therefore, there may be differences in recurrence-suppressing effects when SVR is achieved with an IFN-free DAA regimen vs. an IFN-containing regimen as postoperative adjuvant therapy after HCC treatment. In addition, there have been recent reports indicating that the recurrence rate may be increased after DAA therapy [8,9], as well as reports that contradict this [10–13]. A theoretical background for this increased recurrence rate may be that patients develop an immune dysfunction immediately following HCV eradication [14,15]. This can also be postulated based on reports of HBV re-activation that occurs during or immediately after DAA therapy [16–18]. The disappearance of hepatic lymphocyte infiltration associated with hepatitis eradication from the liver, as well as decreases in endogenous IFN secretion, may be involved in this pathway.

Despite these theoretical concerns about the reduced efficacy of recurrence suppression by IFN-free DAA regimens, the present study showed that the early recurrence rate of HCC was not different between patients who underwent IFN and those who underwent IFN-free DAA treatment. In the current investigation, the IFN-free DAA group was significantly older and included more patients with advanced fibrosis. This indicates that the IFN-free DAA group included more patients with a high risk of HCC recurrence. Nonetheless, there were no differences in the recurrence rate between IFN therapy and IFN-free DAA therapy in SVR patients. This suggests that achieving SVR with IFN-free DAA therapy has a similar or greater effect in suppressing HCC recurrence compared to achieving SVR with IFN therapy. Logistic regression and Cox proportional hazards model analysis identified factors associated with recurrence, such as HCC clinical stage, AFP at completion of antiviral therapy, and duration from last HCC treatment to starting antiviral therapy. These factors reflect the HCC in its original state, as well as after its treatment, indicating that the type of antiviral therapy did not contribute to its subsequent recurrence.

Although previous studies have shown that HCC recurrence was significantly suppressed with IFN in patients who achieved SVR compared to non-SVR patients, the HCC recurrence rate was still high after HCV elimination, indicating that HCV elimination cannot be an endpoint in the treatment of HCV-associated HCC [19–26]. This indicates that there is an extremely high carcinogenic potential in the non-cancerous portion of the liver in hepatitis C patients who have previously developed HCC. HCV elimination may improve liver function, leading to long-term survival in HCC patients, but it is not a sufficient condition for complete suppression of HCC recurrence. Establishing standard measures to suppress HCC recurrence after HCV eradication is desired. Furthermore, basic studies to assure the suppression of HCC recurrence after HCV elimination are also necessary.

While it is important to study the survival rate in patients who have undergone DAA therapy after HCC curative treatment, a sufficient observation period has not yet been achieved. In a previous study during the IFN era in which we assessed patients who had undergone IFN treatment for hepatitis C following HCC treatment, the survival rate was dramatically better in the SVR group than in the non-SVR group [6]. This improvement in survival rate occurred not only because mortality by liver failure was decreased due to improvement of liver function, but also because treatment options for HCC recurrence expanded as a result of improved hepatic reserve capacity, thereby enhancing curability. Therefore, an improvement in the survival rate can be fully anticipated in patients who achieve SVR with DAA therapy. While the survival benefit for patients treated by DAA should be examined in the future, it is primarily necessary to perform careful follow-up for HCC recurrence to achieve improvement of the survival rate.

There are several limitations to this study. First, this was a retrospective cohort study. Second, HCC curability and diagnosis of HCC recurrence were not strictly defined centrally and were determined by the attending physician. Third, because the duration of antiviral therapy differed depending on the regimen, there would have been a bias in either situation, whether the recurrence of HCC was counted from before starting the therapy or whether it was counted from after completing the therapy. However, understanding these limitations, we believe that this study has significant value in that it investigated numerous patients who were treated in actual clinical practice.

In conclusion, the HCC recurrence rate was compared between IFN therapy and IFN-free DAA therapy in patients who previously underwent curative treatment for HCC, and no differences were observed between the two groups. The outcomes of this study indicate that IFN-free DAA therapy can be recommended as an anti-HCV method following HCC treatment.

## Supporting information

S1 Table The clinical data of the study participants. (XLSX)

S2 Table TREND statement checklist. (PDF)

S1 File Observational study protocol. (DOCX)

S2 File Observational study protocol (Original Japanese). (PDF)
