# Supplementary material for: Does interferon-free direct-acting antiviral therapy for hepatitis C after curative treatment for hepatocellular carcinoma lead to unexpected recurrences of HCC? A multicenter study by the Japanese Red Cross Hospital Liver Study Group
Source: PLoS One. 2018 Apr 16;13(4):e0194704. doi: 10.1371/journal.pone.0194704 (PMC5901785; doi:10.1371/journal.pone.0194704)
Supplement: S1 File — (DOCX) [file pone.0194704.s003.docx]

**Observational Study Protocol**

**1. Study Title**

Treatment comparison of oral direct-acting antiviral therapy and interferon-based therapies against hepatitis C virus infection after treatment of hepatocellular carcinoma

**2. Study Administrative Structure**

Principal Investigator: Kouji Joko

Institution: Matsuyama Red Cross Hospital

Department: Center for Liver-Biliary-Pancreatic Diseases

Address: 1 Bunkyouchou, Matsuyama-shi

Phone: 089-924-1111

Email: koujijoko@matsuyama.jrc.or.jp

**3. Study Objective, Purpose and Rational Scientific Reasoning**

Hepatocellular carcinoma (HCC) is a complication of chronic hepatitis C virus (HCV) infection and despite the advances in treatment modalities it remains a disease with a high recurrence rate. The Japan Society of Hepatology’s “Guidelines for the Management of Hepatitis C Virus Infection” recommend the use of interferon (IFN)-based antiviral therapy for chronic HCV infection and cirrhosis patients to prevent carcinogenesis and to improve prognosis by enhancing the hepatic spare ability. The mechanism to prevent recurrence of HCC has been cited as involving IFN i) eradicating HCV, ii) reducing hepatitis infection, and iii) having a direct antitumor effect. The actual mechanism, however, remains unknown. Some studies have reported the ineffectiveness of preventing recurrence^1),2)^. Treatment aiming to achieve sustained viral response (SVR) is given to patients who had been cured of HCC for secondary as well as primary HCC prevention.

In July 2014, a combination therapy with direct-acting antiviral agents (DAA), daclatasvir (an NS5A replication complex inhibitor) and asunaprevir (an NS3 protease inhibitor), was approved in Japan. Since then, numerous IFN-free therapies have begun to emerge. It is an important issue to determine what type of antiviral treatment is best suited in preventing the recurrence of HCC for patients who had undergone HCC treatment and have chronic HCV infection as a complication. The aim of this study is to establish a standardized treatment by investigating patients who had undergone IFN and DAA therapies after HCC treatment to understand the current state of antiviral therapies after HCC treatment. This is a joint research project within the Japanese Red Cross Hospital Liver Study Group.

References

1) Mazzaferro V, et al : Prevention of hepatocellular carcinoma recurrence with alpha-interferon after liver resection in HCV cirrhosis. Hepatology 44 : 1543～54, 2006.

2) Chen LT, et al : Long-term results of a randomized, observation-controlled, phase III trial of adjuvant interferon Alfa-2b in hepatocellular carcinoma after curative resection. Ann Surg 255 : 8～17, 2012.

**4. Study method and period**

◆ Study Method

This is a retrospective study of patients who had previously undergone HCC treatment and received IFN-based or DAA (IFN-free) therapies at the various facilities within the Japanese Red Cross Liver Network. Therapeutic effect and prognosis (recurrence and death) will be investigated through a follow-up study after treatment.

Primary endpoint: Cumulative recurrence rate of HCC

Secondary endpoint: SVR rate and alpha-fetoprotein (AFP) normalization rate

◆ Study Period

September 1, 2014 to March 31, 2018

**5. Subject Selection Policy**

Patients with chronic HCV infection who had undergone curative HCC treatment through surgical resection and radiofrequency ablation that met the following inclusion criteria were selected:

- Patients with HCV infection
- Age 20 or older
- Child-Pugh A classification in cirrhosis patients
- Less than stage III HCC

**6. Informed Consent**

Since this study is a non-invasive, non-interventional observational study that relies on laboratory results of routine check-ups, disclosure and notification of information is conducted in advance based on ethical guidelines of medical research on human subjects. Also, an opt-out method, where subjects are guaranteed the opportunity to refuse participation, is used. For subjects to be able to opt-out of the study, written notices must be displayed and placed in a location that can be easily checked and accessed by the subjects. Furthermore, once a request declining participation is given by the subject, the subject’s medical data must be immediately removed from the analysis and it will not be used in this study.

**7. Handling of Personal Information**

All personal information (name of the patients, date of birth, chart numbers, home address and phone numbers) collected in the medical data is erased, anonymized and linked to a corresponding registered identifier at the Division of Gastroenterology and Hepatology in the Musashino Red Cross Hospital. The password-guarded correspondence table matching the identifiers with the subject’s personal information is stored in a computer with no external connections. This computer is locked in a room where the Principal Investigator is the only person with the keys to control access into and out of the room.

**8. Risk, Responsibilities and Benefits**

There is no direct advantage to the subject by participating in this study. However, it may be possible to contribute to the future advancement of medicine through the study findings. As this is a retrospective study of clinical data, there are no direct disadvantages to the subjects.

**9. Samples, Storage and Disposal of Data**

All the clinical data and records used in this study will be securely stored in a locked storage room under the control of the Principal Investigator, Kouji Joko, at the Center for Liver-Biliary-Pancreatic Diseases in the Matsuyama Red Cross Hospital. The information, such as the registered identifiers, will be deleted and all data and records will be disposed after the termination of the study.

**10. Reporting to the Heads of the Research Facilities**

The progress of the study will be reported in writing to the Heads at least once a year. When evidence or information is gathered that negatively affects or has the potential to affect the ethical validity and scientific rationality of the study, a safety report will be disseminated immediately. When evidence or information is gathered that negatively affects or has the potential to affect the adequacy of the implemented study and the credibility of the study findings, a report such as a non-conformity report will be issued immediately. Cancelation and/or termination notices will be given accordingly. The final study results will be published.

**11. Source of Funding and Conflict of Interest**

Japan Agency for Medical Research and Development (AMED)

There are no conflicts of interest/relationships that could become problematic by implementing this study.

**12. Information Disclosure and Attribution of the Study Results**

We are planning to present the research findings at national/international conferences and publish them as a research paper. All research findings will belong to the Matsuyama Red Cross Hospital. No identifiable personal data will be published.
