# Supplementary material for: Does interferon-free direct-acting antiviral therapy for hepatitis C after curative treatment for hepatocellular carcinoma lead to unexpected recurrences of HCC? A multicenter study by the Japanese Red Cross Hospital Liver Study Group
Source: PLoS One. 2018 Apr 16;13(4):e0194704. doi: 10.1371/journal.pone.0194704 (PMC5901785; doi:10.1371/journal.pone.0194704)
Supplement: S2 File — (PDF) [file pone.0194704.s004.pdf]

日本赤十字社
Japanese Red Cross Society

# 観察研究計画書

## １．研究の名称

肝細胞癌治療後のＣ型肝炎に対するDAAs経口剤とIFNを含む治療の比較

## ２．研究の実施体制

研究責任者：上甲康二
所属組織：松山赤十字病院
所属部署：肝臓・胆のう・膵臓内科
住所：松山市文京町１
電話：089-924-1111
Email：koujijoko@matsuyama.jrc.or.jp

## ３．研究の目的・意義・科学的合理性の根拠

Ｃ型慢性肝炎に合併する肝細胞癌は、治療法の進歩にもかかわらず再発率の高い疾患である。日本肝臓学会の「Ｃ型肝炎治療ガイドライン」では、Ｃ型慢性肝炎や肝硬変に対しインターフェロンを中心とする抗ウイルス療法を行うことは、発癌予防および肝予備能改善になり予後が改善するため推奨されている。一方、肝細胞癌再発抑制の機序としては、IFNによる①HCVの排除②肝炎の鎮静化③直接的な抗腫瘍効果、などが挙げられているが未だに明らかではなく、再発抑制に有効ではないとする報告もある[1),2)]。肝細胞癌根治後の症例では、一次癌予防と同様に二次癌予防を期待して、SVRを目指した治療が行われているが、今後、高いSVR 率が二次癌予防にも有効であるかについて検証する必要があると考えられる。

わが国では2014年7月に、DAA（直接作用型抗ウイルス剤：Direct-acting Antiviral Agents）であるダクラタスビル（NS5A複製複合体阻害剤）＋アスナプレビル（NS3プロテアーゼ阻害剤）併用療法が承認され、その後次々とIFN free治療が始まった。

Ｃ型慢性肝炎に合併した肝細胞癌治療後の症例に対する抗ウイルス療法は、どのような治療が再発抑制に最適か解明することが重要な課題である。そこで、肝細胞癌治療後のIFN療法とDAAs療法の症例調査を行い、肝細胞癌治療後の抗ウイルス療法の現状を把握し、治療を標準化することを目的とした。全国赤十字病院肝疾患ネットワークでの共同研究である。

参考文献

1) Mazzaferro V, et al : Prevention of hepatocellular carcinoma recurrence with alpha-interferon after liver resection in HCV cirrhosis. Hepatology 44 :

1543～54, 2006.
2) Chen LT, et al : Long-term results of a randomized, observation-controlled, phase III trial of adjuvant interferon Alfa-2b in hepatocellular carcinoma after curative resection. Ann Surg 255 : 8～17, 2012.

## ４．研究の方法・期間

◆方法

全国赤十字病院肝疾患ネットワークの各施設において、過去に IFN を含む治療または DAAs (IFN free) 治療を導入された肝細胞癌治療後の患者を retrospective に調査する。治療後は追跡調査を行い、治療効果、予後（発癌、死亡）を調査する。

主要評価項目：肝癌累積再発率

副次評価項目：SVR 率、AFP 正常化率

◆期間：

2014 年 9 月 1 日～2018 年 3 月 31 日

## ５．研究対象者の選定方針

外科手術、ラジオ波焼灼術によって肝細胞癌を根治しえた C 型慢性肝炎・肝硬変症例で、以下の基準を満たすもの。

・HCV 感染患者
・年齢 20 歳以上
・肝硬変症例は Child-Pugh 分類 A とする
・肝細胞癌 stageⅢまで

## ６．インフォームド・コンセント

本研究は、通常診療における検査結果を用いる侵襲を伴わず介入を行わない観察研究であるため、人を対象とする医学系研究に関する倫理指針に則り、あらかじめ情報を通知・公開し研究対象者等が拒否できる機会を保証する方法（オプトアウト）とする。オプトアウトによる場合は、研究対象者等が確認できる場所への書面の掲示・備え付け等により、研究対象者等が容易に知り得る状態に置く必要がある。なお、本研究の被験者になることを希望しない旨の申し出があった場合は、直ちに当該被験者の診療情報を解析対象から除外し、本研究に使用しないこととする。

## ７．個人情報の取扱い

採取した診療情報は、武蔵野赤十字病院消化器科において、患者氏名、生年月日、カルテ番号、住所、電話番号を消去し、代替する登録番号にて連結可能匿名化する。登録番号と被験者個人を連結する対応表は、外部と接続できないパソコンで管理し対応表の

ファイルにはパスワードを設定する。このパソコンを設置する部屋の鍵は研究責任者のみが保有しており、入退室を管理する。

## ８．リスク・負担と利益

本研究により被験者が直接受けることができる利益はない。しかし、研究成果により将来の医療の進歩に貢献できる可能性がある。また、本研究は臨床情報を用いた後向き研究であり、被験者に直接的不利益は生じない。

## ９．試料・情報の保管・廃棄

本研究における臨床情報等は、松山赤十字病院肝臓・胆のう・膵臓内科において、研究責任者上甲康二の責任の下、施錠可能な保管庫に厳重に保管する。研究が終了すれば、登録番号等を消去し廃棄する。

## １０．研究機関の長への報告

少なくとも年に１回、研究の進捗状況を長に文書で報告する。研究の倫理的妥当性や科学的合理性を損なう事実もしくは情報、または損なうおそれのある情報を得た場合は、速やかに安全性情報に関する報告を行う。研究実施の適正性や研究結果の信頼性を損なう事実もしくは情報、または損なうおそれのある情報を得た場合は、速やかに不適合等報告書を提出する。中止・終了報告は適宜行う。成果の最終の公表を報告する。

## １１．資金源・利益相反

日本医療研究開発機構（AMED）

本研究の実施に際し、特に問題となる利益相反関係はない。

## １２．研究に関する情報公開の方法・研究成果の帰属

本研究の成果は国際・国内学会発表及び論文発表を予定している。また、研究成果は全て松山赤十字病院に帰属する。その際、個人を識別できる情報は一切含まない。
